# Architecture of severe fever with thrombocytopenia syndrome virus

**DOI:** 10.1093/procel/pwad019

**Published:** 2023-04-11

**Authors:** Zixian Sun, Jing Cheng, Yuan Bai, Lin Cao, Daoxin Xie, Fei Deng, Xinzheng Zhang, Zihe Rao, Zhiyong Lou

**Affiliations:** Department of Basic Research, Guangzhou Laboratory, Guangzhou 510005, China; MOE Key Laboratory of Protein Science, School of Medicine, Tsinghua University, Beijing 100084, China; National Laboratory of Biomacromolecules, CAS Center for Excellence in Biomacromolecules, Institute of Biophysics, Chinese Academy of Sciences, Beijing 100101, China; University of Chinese Academy of Sciences, Beijing 100049, China; National Virus Resource Center, Wuhan Institute of Virology, Chinese Academy of Sciences, Wuhan 430071, China; State Key Laboratory of Medicinal Chemical Biology and College of Life Science and Pharmacy, Nankai University, Tianjin 300350, China; MOE Key Laboratory of Protein Science, School of Medicine, Tsinghua University, Beijing 100084, China; University of Chinese Academy of Sciences, Beijing 100049, China; National Virus Resource Center, Wuhan Institute of Virology, Chinese Academy of Sciences, Wuhan 430071, China; University of Chinese Academy of Sciences, Beijing 100049, China; National Laboratory of Biomacromolecules, CAS Center for Excellence in Biomacromolecules, Institute of Biophysics, Chinese Academy of Sciences, Beijing 100101, China; Department of Basic Research, Guangzhou Laboratory, Guangzhou 510005, China; MOE Key Laboratory of Protein Science, School of Medicine, Tsinghua University, Beijing 100084, China; State Key Laboratory of Medicinal Chemical Biology and College of Life Science and Pharmacy, Nankai University, Tianjin 300350, China; MOE Key Laboratory of Protein Science, School of Medicine, Tsinghua University, Beijing 100084, China

**Dear Editor,**

Severe fever with thrombocytopenia syndrome (SFTS) is an acute infectious disease caused by severe fever with thrombocytopenia syndrome virus (SFTSV) and is characterized by rapid onset, high mortality and widespread distribution. The fatality rate can be as high as 10%–30%, which is significantly higher than that of the hemorrhagic fever caused by Hantavirus in the same area (Song et al., 2018). However, no effective treatments or vaccines are available yet, and the underlying pathogenic mechanisms of the virus are poorly understood.

SFTSV has three single-stranded negative-sense RNA segments (L, M, and S), and belongs to the *Phenuiviridae* family within the *Bunyavirales* order (Wang et al., 2021). As is known to all, the structure study of such a huge enveloped virion is a big challenge. At present, most of the enveloped viruses that have been analyzed contain the capsid shell in addition to the glycoprotein-bound envelope, such as dengue virus (DENV) (Prasad et al., 2017). The smaller diameter or presence of scaffold proteins can improve the defocus refinement and the stability of the virions, which helps obtain high-resolution structures. However, the virus shell of SFTSV consists of only lipid bilayer envelopes covered with capsomers composed of transmembrane glycoproteins (Gc and Gn) that lack a capsid. Moreover, the tomographic characterization of purified native virions by pioneering researchers showed that although the virions were uniform in size, they often deviated from their spherical shape, and the particles became majorly deformed. In previous studies, it has been impossible to obtain high-resolution structures of viruses related to the Bunyavirales order, including Tula virus (TULV) and Rift Valley fever virus (RVFV) (Halldorsson et al., 2018; Serris et al., 2020). These studies revealed that the glycoprotein shell of bunyavirus has a similar overall architecture but displays some unique features as well, suggesting the structural diversity of the Bunyavirales order. However, these densities are not clear enough to trace the folding of the polypeptides to assemble the virus particle. This limits our understanding the assembly and pathogenesis of bunyavirus.

In this study, we first used traditional virus purification methods to purify the cultured virus (see Supplementary Materials). However, most of the particles are distorted because they squeeze together and even fragment, causing the internal genome to be released (Fig. S1A), possibly as a result of the purification procedure. To acquire the stable and intact virions for cryo-EM analysis, we explored and optimized the virus purification method (see Supplementary Materials). By adjusting the ratio, concentration and fixation time of the stabilizing solution, we made good initial progress (Fig. S1B). Considering that too many centrifugation steps will increase mechanical damage and cause serious particle fragmentation, we adjusted the viral culture conditions to obtain high-quality products that can not only improve viral infection and packaging efficiency but also reduce impurities in order to simplify purification. Finally, we only used sucrose-cushion ultracentrifugation and obtained intact virus particles with good uniformity, suitable concentration and stable (Fig. 1A).

**Figure 1.**
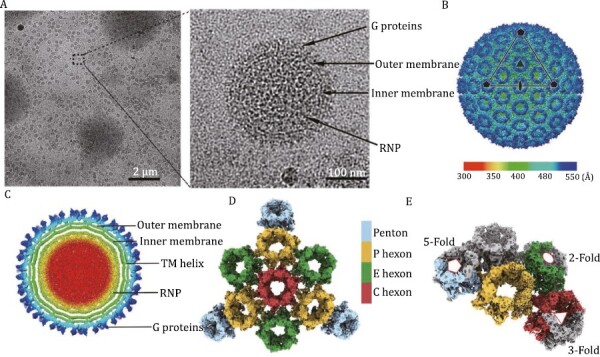
**Overall structure of the SFTSV.** (A) Cryo-EM of the purified SFTSV virion containing four structural layers. (B) Density map of the icosahedral SFTSV glycoprotein shell, colored by radius with the scheme below. An icosahedral facet is indicated by a triangle. The 5-fold, 3-fold, and 2-fold axes are denoted by pentagons, triangles, and ovals, respectively. (C) Central slice of the icosahedral SFTSV virion structure. The glycoproteins, outer membrane, inner membrane, transmembrane helix and RNP are labeled. (D) Magnified view of one facet of the icosahedral glycoproteins with structural components differentially colored. (E) Density map of an ASU, colored by protein. The G proteins in different capsomers are colored in the same scheme as used in (D).

Following the process mentioned above, we imaged the intact virions by electron-counting cryo-EM, and an overall resolution of 8 Å was obtained using the conventional icosahedral averaging method with 11,187 virion particles binned by 4 (Fig. S2). To further counteract the effects of conformational heterogeneity within the large capsid particle, we optimized the reconstruction by using the “block-based” reconstruction (BBR) and *in situ* single particle analysis (isSPA) methods. Sub-particles around the 3-fold, 2-fold and 5-fold axes were re-extracted for BBR. Within the local refinement on each region, we obtained a reconstruction of the 3-fold axis region with a resolution of 5.6 Å and a reconstruction of the 2-fold axis region with a resolution of 5.9 Å. While the 5-fold axis region exhibited the most significant conformational heterogeneity, probably also because this region is an internal genomic release site (Liu et al., 2019), it showed a relatively low resolution with a local resolution of approximately 7.2 Å. The BBR method relied heavily on the initial translational and rotational parameters obtained from the icosahedral reconstruction. However, the structure of SFTSV may deviate largely from the symmetric structure as indicated by the 2D class averages. Thus, these parameters of many of the virus particles from a refinement with icosahedral symmetry were incorrect, which led to a limited resolution of the reconstruction from the BBR method. To avoid using these parameters and further improve the local resolution, we processed the 3-fold axis region and 2-fold axis region with isSPA methods. Using maps generated by BBR as templates, we re-searched globally the locations and orientations of the sub-particles with isSPA. Finally, the resolution of the 3-fold proteins was improved to 4.5 Å, and the resolution of 2-fold proteins was pushed to 5.18 Å (Figs. S2 and S3), respectively. The combinational techniques used in study will provide a successful example for the structure study of huge enveloped viruses.

At the same time, consistent with earlier reconstructions at subnano resolutions (Halldorsson et al., 2018), our results show that SFTSV is constructed of 110 hexons and 12 pentons arranged in a triangulation number (*T*) = 12 icosahedral particle and a diameter of approximately 110 nm (Fig. 1B). SFTSV is a segmented negative-sense RNA virus. The three genome segments encode four structural proteins. Among them, the nucleoproteins (N protein) enwrap each genomic viral RNA (vRNA) and interact with the single-polypeptide polymerase (L protein) form the ribonucleoprotein complexes (RNPs), which are responsible for the transcription and replication of the vRNA. The RNPs occupy this volume (~350 Å in diameter) as densely packed, thread-like structures, filling the interior volume (Fig. 1C). The outer rim of the particles is occupied by spikes (Fig. 1C, arrows), which we assign to viral glycoproteins. Beneath the spike layer is a continuous layer, which we assign to the lipid bilayer (Fig. 1C). The glycoproteins were clustered as torch morphological units, and the 720 Gn–Gc heterodimers formed the glycoprotein shell (Fig. 1B and 1C). The glycoprotein shell was organized into three types of quasi-equivalent hexons (peripentonal, edge, and central) and pentons (Fig. 1D). An asymmetric unit (ASU) consists of 12 copies of Gn–Gc dimers, which exist in one P-Hex, one-third of a C-Hex, one-half of an E-Hex and one-fifth of a Pen (Fig. 1E). Because SFTSV lacks the matrix protein or capsid protein, the glycoproteins are responsible for the structural stability of the virus, and interspike contacts likely have a major role in determining virion packing.

SFTSV and other bunyaviruses use their glycoproteins for entry into target cells and assembly of progeny particles in infected cells. In addition, glycoproteins, as the only proteins exposed on the surface of the virus, are also protective antigens of the virus, so they have become a key target for antibody design (Zhu et al., 2018). The M segment of the virus encodes a polyprotein precursor that is sequentially cleaved by cellular proteases during transport to the endoplasmic reticulum (ER) to become mature two glycoproteins, Gn and Gc (Fig. 2A). At present, the separate extracellular crystal structures of the ectodomain of Gn and Gc have been resolved (Halldorsson et al., 2016; Wu et al., 2017), but they act as a complex on the surface of the virus. There is no clear conclusion about the way the complex is assembled in the native state, and the structures and interactions of the glycoproteins in the virion remain unclear. According to our density map, we confirmed the molecular details of the native SFTSV Gn–Gc pre-fusion heterodimer (Figs. 2B, S4A and S6; Table S1).

**Figure 2.**
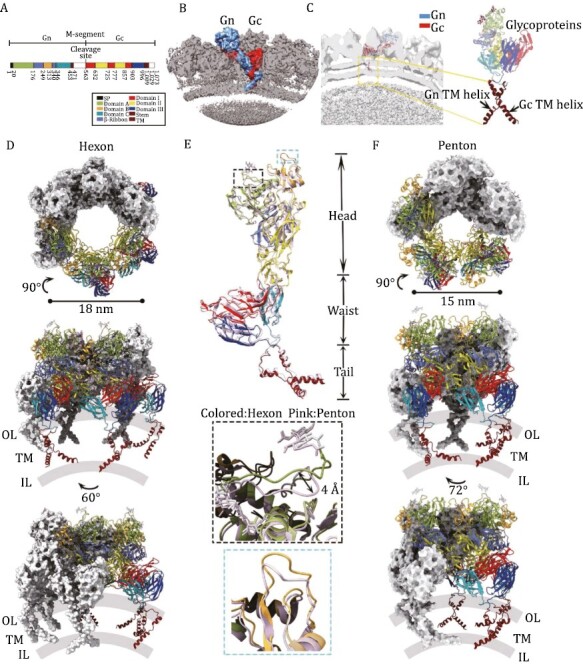
**Organization and atomic structure of native SFTSV glycoproteins.** (A) Schematic representation of the full M segment of SFTSV containing Gn and Gc with the cryo-EM structure colored by domain: domain A in chartreuse; domain B, gold; domain C, light sea green; β-ribbon, cornflower blue; domain I, red; domain II, yellow; domain III, blue; stem peptide, chocolate; and transmembrane helix, crimson. Domains are not modeled and colored white, and the signal peptide is colored black. (B) Native SFTSV Gn–Gc pre-fusion heterodimer displayed on the virus particles. Gn colored sky blue, Gc colored red. (C) The density map of the transmembrane region of SFTSV is specially marked with a yellow dotted box. (D) Top view and side view of the overall structure of a hexon. Three adjacent Gn–Gc heterodimers are shown as cartoon models, and the other three are shown as gray surface models. Special interhexamer interactions are marked with magenta dashed boxes. Outer (OL) and inner (IL) leaflets of the lipid bilayer are coloured in semitransparent gray and transmembrane region (TM) are labeled. (E) Superposition of the glycoprotein conformers in hexon and penton capsomers. The inset shows close-up views of distinguished structural motifs with different conformations. (F) Architecture and interprotomer interactions of a penton, shown in similar views and color schemes to those of the hexon in (D).

The N-terminal ectodomain of Gn binds to the cell surface, and the C-terminal transmembrane helix anchors to the viral membrane. The ectodomain can be divided into the head and stem domains. The structure of the head domain has been resolved (Wu et al., 2017). Similar to the previously reported crystal structure results, the Gn head domain is folded into a similar triangular structure and consists of three subdomains, namely, domain A and domain B, and a β-ribbon connector (Figs. 2E, S4 and S7). In addition, the C-terminal third domain of Gn of Phlebovirus was absent in previous studies. In this study, domain C is folded into a 7-stranded β-sheet-rich structure, with a size similar to that of domain C of alphavirus E2 (Fig. 2E). Domain C of Gn contacts domain III of Gc near the viral membrane. Together with domain A, domain C is involved in threefold contacts stabilizing the trimeric spike (Guardado-Calvo and Rey, 2017). Gc is a fusion protein facilitating virus entry into host cells. Moreover, it responds to the reduced pH of endocytic compartments with a conformational change. Here, we describe the structure of Gc in its pre-fusion conformation. Gc from SFTSV was recently crystallized at acidic pH in its post-fusion state (Halldorsson et al., 2016). Therefore, we used SFTSV Gc in the post-fusion state (PDB ID: 5G47) and RVFV Gc in the pre-fusion state (PDB ID: 4HJC) as guidance to build the model. Similar to Gn, Gc can also be divided into extracellular regions and C-terminal transmembrane regions. The extracellular region can be divided into three subdomains: domain I, domain II, and domain III (Figs. 2E and S4). Moreover, domain I and domain III of Gc differ greatly from the previously reported crystal structures (Fig. S7).

Gn and Gc assemble into a heterodimer on the viral surface, with Gn forming a capsid spike and the Gc portion located below, closer to the lipid membrane (Fig. 2B). The surface proteins are organized in a unique intertwined conformation between the spikes. The structure shows that the Gn–Gc heterodimer has the shape of a duplex-like plate approximately 165 Å long, 95 Å wide and 53 Å thick and comprises a reclining head, barrel-shaped waist and crossed tail (Fig. S4A). To understand how virion assemble shell in the final life cycle, we attempted to understand the high-order assembly of the Gn–Gc spike complex on the virion surface. The reclining head consists mainly of the head domain of Gn and domain II of Gc, which maintains the interaction networks of both intra- and inter-hexon/penton capsomers (Fig. S5; Tables S3, S4 and S5). Moreover, as in the varicella-zoster virus (VZV), an N-terminal loop forms a lasso (N-lasso) at domain B to fasten to the adjacent hexon/penton capsomers. The ectodomains of Gn–Gc are divided into two parts (i.e., the head domain and the stem domain), both of which participate in the assembly of individual penton and hexon capsomers (Fig. 2D and 2F). We next compared the difference between the Gn–Gc dimers on hexamers and pentamers. From the overall layout, the spikes at the pentavalent positions are slightly smaller (~15 nm in diameter) than the spikes at other positions (~18 nm in diameter) (Fig. 2D and 2F). This assembly difference and curvature change between hexamers and pentamers cause some variation in the mutual positions of Gn and Gc between neighbors as well as in the amino acids of the interaction surface (see Supplementary Materials). The structural information observed in our structure presents a most native state of virus in nature and the most convinced state to understand the modification of viral envelop proteins. Hence, we summarized the currently reported phlebovirus-related antibodies or epitopes and presented them separately in the structures (Fig. S4B). The mapping of the epitopes of the reported neutralizing antibodies on Gn and Gc clearly suggests conformational shifting occurring in virus entry, providing useful information to neutralizing antibody development in the future (Fig. S4). Through comprehensive data analysis, we found that the organization of Gn–Gc spikes on the SFTSV virions did not show the pre-fusion and post-fusion conformation at the same time as SARS-CoV-2 virions (Yao et al., 2020). All spikes were in the pre-fusion states (Fig. 1A and 1B). This observation suggests that SFTSV spikes are steady on the viral envelope and that some antibodies function at the post-attachment step and inhibit subsequent conformational rearrangements in the Gn–Gc interface (Chapman et al., 2021).

Bunyaviruses contain neither a matrix protein nor a capsid protein, so the cytoplasmic tail (CT) domain of one or both viral glycoproteins is presumed to be involved in the assembly and budding processes. Recent studies have demonstrated that the CT domain can interact directly with nucleoproteins, thereby facilitating the packaging of the RNPs into particles to prevent the release of empty particles (Guo et al., 2012). However, the structures of the terminal transmembrane helices of Gn–Gc proteins and the mechanism for these helices anchoring Gn–Gc to the double-membrane to assemble the virus particle are not reported previously.

The glycoprotein shell densities have some tubular shapes corresponding to helices present in previous studies (Kostyuchenko et al., 2013; Heinz and Stiasny, 2017). We speculate that the structure of the helices is the CT domain of SFTSV (Fig. 2C). However, the density of the transmembrane helices compared with that of the extracellular region was slightly weaker, as the transmembrane helices disappeared at lower visualization thresholds than the extracellular helices (Fig. 2C). Helix density for this structure is poor, indicating either inherent flexibility of the transmembrane region or different relative positions in individual particles. Because the RNP is packed very densely, the increase in resolution is limited by the signal-to-noise ratio. Due to the quality of the density, we placed poly-Ala in the transmembrane region without side chain information. To optimize the fit, individual helices were allowed to move independently to a small degree, to better fit the experimentally obtained density map. The real-space refinement was subsequently used to ensure realistic bond lengths and geometry. Finally, we obtained the structural model of the TM helix (Fig. 2C and 2E).

Both the CT domain of Gn and the CT domain of Gc are anchored to the viral membrane by two transmembrane (TM) helices. Approximately 20 residues in our density map formed a helix clamp at the base of the adjacent stem region, and the helices were connected with the stem region by flexible loops (Figs. 2C and S6). The helix of Gn and the helix of Gc cross and overlap near the membrane area, resulting in a more compact glycoprotein shell in SFTSV between the lipid bilayer and RNP. However, due to the limitation of resolution, the CT domain could not be fully resolved, with approximately 100 residues missing in our density map, suggesting the inherent flexibility of the membrane protein complex. This spacing also reflects the extensive contacts seen between the membrane and the RNP (Figs. 1C and 2C). Therefore, the vRNA packaging mechanism of SFTSV remains to be determined.

In our study, we present molecular insights into the structures of glycoproteins in pre-fusion and how they assemble on authentic viruses. We also analyzed the detailed availability of epitopes. The structure of SFTSV is the current largest solved structure at near-atomic resolution among the complete living virions of enveloped viruses. It provides a complete and detailed picture to understand the assembly and the biology of bunyavirus. Furthermore, this study provides a structural basis for the rational design of vaccines or antiviral drugs.

## Supplementary Material

pwad019_suppl_Supplementary_Material

## Footnotes

Density maps, structure factors and atomic models that support the findings of this study have been deposited in the Electron Microscopy Data Bank (EMDB) and in the Protein Data Bank (PDB) with the accession codes PDB: 7X6U (Gn–Gc of SFTSV 3-fold hexamer), EMD-33027 (SFTSV 3-fold hexamer) and PDB: 7X6W (Gn–Gc of SFTSV 2-fold hexamer),EMD-33029 (SFTSV 2-fold hexamer) and PDB: 7X72 (Gn–Gc of SFTSV 5-fold pentamer), EMD-33030 (SFTSV 5-fold pentamer).

This work was supported by grants from the National Program on Key Research Project of China (2018YFE0200402), the National Natural Science Foundation of China (32188101 and U20A20135), the National Program on Key Research Project of China (2020YFA0707500), and Tsinghua University Spring Breeze Fund.

Z. L., and Z.R. conceived the project and designed the experiments. F.D., Y.B., and Z.S. performed virus cultivation and purification. Z.S., and L.C. prepared the cryo-EM grids and collected cryo-EM data. X.Z., J.C., and Z.S. carried out data processing and reconstruction. Z.S., Z.L., and D.X. analyzed the data. Z.L., and Z.S. wrote the manuscript. All authors discussed the experiments, read, and approved the manuscript.

All the authors declare no conflict of interest.
