# Supplementary material for: Architecture of severe fever with thrombocytopenia syndrome virus
Source: Protein Cell. 2023 Apr 11;14(12):914–8. doi: 10.1093/procel/pwad019 (PMC10691843; doi:10.1093/procel/pwad019)
Supplement: pwad019_suppl_Supplementary_Material [file pwad019_suppl_supplementary_material.pdf]

Supplementary Materials

Architecture of severe fever with thrombocytopenia syndrome virus

Zixian Sun[1,2,$], Jing Cheng[4,$], Yuan Bai[3,5,$], Lin Cao[6], Daoxin Xie[2], Fei Deng[3,5,*], Xinzheng Zhang[3,4,*], Zihe Rao[1,2,6,*], Zhiyong Lou[2,*]

[1] Department of Basic Research, Guangzhou Laboratory, Guangzhou 510005, China

[2] MOE Key Laboratory of Protein Science, School of Medicine, Tsinghua University, Beijing 100084, China

[3] University of Chinese Academy of Sciences, Beijing 100049, China

[4] National Laboratory of Biomacromolecules, CAS Center for Excellence in Biomacromolecules, Institute of Biophysics, Chinese Academy of Sciences, Beijing 100101, China

[5] National Virus Resource Center, Wuhan Institute of Virology, Chinese Academy of Sciences, Wuhan 430071, China

[6] State Key Laboratory of Medicinal Chemical Biology and College of Life Science and Pharmacy, Nankai University, Tianjin 300350, China

[$] These authors contribute equally to this work

*Correspondence: df@wh.iov.cn (F. Deng), xzzhang@ibp.ac.cn (X. Zhang), raozh@tsinghua.edu.cn (Z. Rao); louzy@mail.tsinghua.edu.cn (Z. Lou);

Contents

Supplementary Information and Methods

Supplementary Figures

Supplementary Tables

References

Supplementary Information and Methods

Viral assembly

The Gn–Gc spike complex forms a six-membered ring through protein-protein interactions. Observation of the top view suggests significant deviations from similar six-fold symmetry in the hexamers, like a garland. (Fig. 2D). On top of each hexon, six Gns form a cap to cover the upper crown and to bridge adjacent Gcs to secure the hexameric structure (Fig. 2D). In particular, the Gn–Gc spike complex forms pairs. Residues in the Gn loop (E151-C156) are stabilized by strong salt bridge interactions with the Gn loop adjacent to the right side (R281, E297-K302, R304) (Table S3). This interaction network helps to stabilize the contacting interface of the upper crown of adjacent Gn-Gc dimers, thereby enhancing the stability of the hexons. Residues in the Gc domain I interact with the β-ribbon region of the adjacent Gn on the right. In addition, hydrophobic interactions between domain II of Gc and domain II of the adjacent Gc on the right (Table S3) cause them to form a tight heterotetramer. Instead, the distance between the adjacent left heads was larger than that of the right heads, which implied that the interaction between domain II of Gc at the waist may play an important role in virion stabilization and assembly (Fig. 2F). Within a different molecular context, the interaction patterns at the interface between penton and hexon capsomers are substantially different. On top of each penton, a large cliff is formed between two adjacent Gns, resulting in few contacts between the stem and head domains (Table S4). The two adjacent side chains form hydrogen bonds between domain I and domain II of Gc to stabilize the pentamer (Table S4). This observation partially explains the more severe conformational flexibility of pentons compared with hexons, as revealed by the cryo-EM densities.

Comparing the Gn-Gc dimers in pentons and hexons, we found that the barrel-shaped waist could be very similar, except for these parts, which revealed distinct conformations in the two capsomer forms (Fig. 2E). The most remarkable difference between the Gn-Gc dimer of penton and hexon conformers lies in the reclining head, which adapts to the different assembly networks with 5-fold or similar 6-fold symmetry, respectively. The loop near the N-linked glycan on the hexamer is pushed away from the envelope surface by ~4 Å relative to the pentamer, but the modification positions of the N-linked glycan are roughly the same. This movement indicates that there may be no site preference when phlebovirus enters cells via interaction with the viral oligo-type glycans. In another region, the loop of hexon is pulled upwards from the viral surface compared with the penton. These movements show that the head of the pentamer is looser than that of the hexamer and that the hexamer pack is stable (Figs. 2D, 2E, and 2F). The loops in the hexons intersect tightly

with neighboring molecules to strengthen the stability of the hexon spikes (Fig. 2D). Owing to the distinct assembly patterns in the pentons and hexons, within the crossed tail, the transmembrane helix and the loop of the stem region in the hexons all undergo a slight shift relative to their counterparts in the pentons. This conformational heterogeneity might allow the capsid to counteract the different pressures exerted on the icosahedral vertices and facets by the packed viral genome.

To further explore the mechanism of SFTSV assembly, we analyzed the interaction between an asymmetric unit composed of 12 Gn-Gc dimers (Fig. S5A). The assembly of penton and hexon capsomers is mediated mainly by the barrel-shaped waist, which forms an enormous contacting network to stabilize the icosahedral cage of the capsid (Fig. S5A). This interaction network involves a few different contact modes at specific sites with distinct molecular contexts. Between adjacent hexons, the waist forms symmetric contact profiles involving two or four Gn-Gc dimers, as exemplified by the E- and P-hexons. The two strands in domain III of the E1 dimer and P5 domain C have hydrophobic interactions (Fig. S5B and Table S5). The other side is just the opposite, a direct interaction exists between the two strands in domain III of the P4 dimer and domain C of E2 (Fig. S5C and Table S5). The C1 and P3 protomers and the E2 and C2 protomers form a similar interaction profile from the side (Figs. S5D, S5E, and Table S5). The network interactions of the penton and its surrounding P-hexons are different from those of other hexons (Figs. S5F, S5G, and Table S5) in two ways. First, Pen1 interacts with two Gn-Gc dimers at the same time. The loop on domain I of Pen1 stretches out to form hydrogen bonds with the loops on domain I and domain III on P6. The other side interactions are remarkably similar to the interactions between hexons. These interactions highlight the central role of domain C and domain III, which contribute to extensive contact networks with various domains of adjacent Gn-Gc dimers.

The potential neutralization mechanism of SFTSV

The study of antibodies as major immune effectors in preventing and treating viral infections has always been highly valued (Crowe, 2017). We aim to further our understanding of the molecular basis of antibody neutralization of viruses and identify key viral epitopes to aid in the development of vaccines and therapeutics against SFTSV infections. Here, we summarize the currently reported phlebovirus-related antibodies or epitopes and present them separately in the structures that we obtained for their summary analysis. These include the following antibodies/epitopes: (1) The only human neutralizing monoclonal antibody Mab4-5 of the Gn-Fab complex structure in SFTSV has been reported (Wu et al., 2017). (2) Antibody Ab10 was isolated from a phage-displayed antibody library, which was constructed from patients infected with SFTSV who had recovered. Furthermore, its possible conformational epitopes were predicted using crosslinking coupled mass spectrometry and by testing its reactivity to

alanine mutants (Kim et al., 2019). (3) Five fine epitopes were obtained using the modified biosynthetic peptide method to map linear B cell epitopes (BCEs) on the Gn from SFTSV. Since one of these epitopes overlaps with the known Mab 4-5, it is not repeatedly marked out in our structure (Moming et al., 2021). (4) Previous structural analysis of Gn-Mab complexes in RVFV has identified a number of neutralizing epitopes, which are scattered around three major patches (A, B and C) (Wang et al., 2019). (5) Some mAbs from the B cells of immune individuals appear following natural infection by RVFV or vaccine. Because the epitopes largely overlap, they are also divided into four major groups (A, B, C and D) (Chapman et al., 2021). However, some specific regions only mapped to some important key amino acids but did not have well-defined sites (Fig. S4B).
Antibodies may exert their neutralizing activities by targeting different stages of viral infection, including blocking viral attachment to its receptors and interfering with viral uncoating, by overstabilizing or destabilizing the virus (Dong et al., 2017; Zheng et al., 2019; Wang et al., 2020). Furthermore, neutralizing antibodies have been shown to target determinants around the fusion loop of the envelope in enveloped viruses. In enveloped viruses, fusion inhibition is a second major mechanism of neutralization (Kim et al., 2019). Although no specific host cell receptor has been identified in this genus thus far, except for DC-SIGN, analysis of the available results suggests that the receptor binding site may be present near Gn domain A or domain B. Like Mab 4-5 and/or E3, these exposed sites on the viral surface block the binding sites or induce steric clashes to prevent viral attachment to the receptors (Wu et al., 2017; Moming et al., 2021). Furthermore, the binding of the antibody to the virus results in the inability of the surface proteins of the virus to undergo dramatic structural rearrangements, thus preventing the triggering of virus uncoating (Dong et al., 2017).

Methods

Cell culture and virus production

African green monkey kidney cells (Vero; ATCC, CCL-81) were cultured in Dulbecco's modified Eagle's medium (DMEM; Sigma, USA) supplemented with 10% fetal bovine serum (FBS; Gibco, Australia) at 37 °C with 5% CO_2 in T75 flasks. SFTSV strain HBMC5_human_2016 (Accession No: CSTR: 16533.06. IVCAS 6.6311) was provided by Professor Fei Deng from the National Virus Resource Center (NVRC) of Wuhan Institute of Virology, Chinese Academy of Sciences.
The Vero cells were seeded for one day until they reached approximately 65-75% confluence, and the medium was replaced with fresh DMEM with 2% FBS containing penicillin (100 U/mL) and streptomycin sulfate (10 μg/mL). The cells were infected with 200 μL SFTSV at 7.96×10^7 TCID50, and the supernatants were harvested 6 days postinfection (d.p.i.) after low-speed

centrifugation. Subsequently, the time for collecting the supernatant was adjusted to 4 d.p.i. Viral titers were measured using the Vero cells in end-point dilution assays as previously described (Yu et al., 2011).

Virus purification

After 6 d.p.i, the cell mediums were harvested and centrifuged at 5,000 × g for 10 minutes at 4 °C to remove large cell debris. Subsequently, the supernatant was purified through a sucrose cushion by transferring it into a Type 45 Ti rotor (Beckman). 2 mL of 20% (w/v) sucrose was gently added to the bottom of the supernatant, followed by centrifugation at 100,000 × g for 1.5 h. The sucrose was removed from the pellet, and 500 µL of PBS buffer was added to cover the pellet, which was kept overnight at 4 °C to allow the pellet to soften. The supernatant was purified with a discontinuous 20% to 60% (w/v in PBS) sucrose gradient and centrifuged at 220,000 × g for 4 h. Fractions with SFTSV particles were collected and dialyzed against the PBS solution. However, as a consequence of this process, most of the particles were distorted because they squeezed together and even fragmented, causing the internal genome to be released (Fig. S1A). We explored and optimized the virus purification method. Previous studies have shown that structural comparisons of live and fixative-inactivated virus particles reveal unremarkable conformational changes, and that fixative treatment is a safe way to maintain the native structure during inactivation. We devised an improved sample preparation strategy based on particle fixation with different fixatives through dialysis, followed by sucrose gradient purification. By adjusting the ratio, concentration and fixation time of the stabilizing solution, we made good initial progress. After 4 d.p.i., the supernatant was spun down at 3,000 × g for 10 min at 4 °C to remove cell debris. The supernatant was stabilized by the addition of 0.05% (w/v) formaldehyde and incubation at 4 °C for 2 d. After 2 days, 0.5% (w/v) glutaraldehyde was added to the virus with incubation at 4 °C for 20 min, and then, 20 mM Tris 7.4 buffer was added to stop this reaction. Then, 10 mL of 20 % (w/v) sucrose cushion was gently added to the bottom of the suspension, and it was centrifuged with an SW32 rotor (Beckman) at 20,000 ×g for 1.5 h. The tube was inverted to remove the sucrose, and the excess solution was wicked off. The pelleted viruses were resuspended in 50 µL of PBS (Gibco, pH 7.4). It was kept overnight at 4 °C to allow the pellet to soften. The purified SFTSV particles were imaged with negative staining EM and Cryo-EM.

Negative stain

For examination by negative staining, preparations of samples were diluted in 1×PBS to a suitable concentration, applied to freshly glow-discharged carbon-coated copper grids, and stained with 2% uranyl acetate. All samples were examined on a Tecnai T12 electron microscope (FEI) equipped with an

LaB6 filament and operated at an acceleration voltage of 120 kV.

Sample preparation and Cryo-EM data collection

Aliquots of 3 µL of purified SFTSV were applied to a fresh glow-discharged holey carbon-coated copper grid (Quantifoil Cu R2/1 + 2 nm C, 200 mesh) and flash-frozen in liquid ethane using an FEI Vitrobot mark IV. Cryo-EM data were collected manually using the SerialEM program in a 300-kV FEI Titan Krios electron microscope operated at a nominal magnification of 130,000× and a pixel size of 1.08 Å per pixel with defocus values from −2.0 to −2.5 µm. A K2 direct detector was used for image recording under super-resolution mode. Each exposure was performed with an accumulative dose of 50 $e^{-}Å^{-2}$, which was fractionated into 36 frames for each image stack. Data collection and refinement statistics are summarized in Supplementary Table 1.

Image processing

A simplified flowchart of the procedure for image processing of SFTSVs is presented in Fig. S2. We manually collected 14,458 raw movie micrographs. The MotionCorr2 program (Li et al., 2013) was used for motion correction. After whole-image CTF estimation using CTFFIND4 (Mindell and Grigorieff, 2003), 14,446 good micrographs were manually selected from the dataset. Particles were picked manually using RELION-2.1 (Scheres, 2012). A total of 23,950 particles from ~10,000 micrographs were extracted for 2D classification, and 17,255 particles were selected for 3D reconstruction. The re-extracted particles were subjected to 3D refinement in I3 symmetry, which resulted in a map at 8 Å.

To compensate for the limitation in flexibility, sub-particles around the 3-fold, 2-fold and 5-fold axes were re-extracted and 2x binned (2.16 Å/pixel) as 3 blocks, and each block was refined and reconstructed separately. The initial orientation and location parameters for the blocks could be calculated based on icosahedral symmetry virus reconstruction. The defocus values of the subparticles were corrected to correct the Ewald sphere effect. After nonalignment 3D classification, approximately 149,484, 281,344 and 118,467 sub-particles were selected for the 3-fold, 2-fold and 5-fold axes, respectively. A local search for better rotation and translation parameters of each selected sub-particle was performed, and the resolution of the 3 blocks reached 5.6 Å (3-fold), 5.9 Å (2-fold) and 7.2 Å (5-fold), respectively. To further improve the resolution, the 5.6 Å and 5.9 Å blocks were used to produce 2D references for global detection of these blocks on 11,187 images of viral particles by isSPA. The densities in a frequency range of 1/100 $Å^{-1}$ to 1/8 $Å^{-1}$ were involved in the calculation. A total of 1,050,900 detections for the 3-fold block and 1,047,057 detections for the 2-fold block were stored for later processing, respectively. For calculation of the 2-fold block, 1,047,057 sub-particles were extracted and 2x binned, 109,076 of which were selected by nonalignment 3D classification. After a local refinement, a reconstruction of 6.2 Å was achieved, which was

worse than the result of BBR. Subsequently, the isSPA sorting algorithm was used to score these sub-particles using frequencies from 1/8 $Å^{-1}$ to 1/6 $Å^{-1}$, from which 54,378 were selected, resulting in a resolution of 5.9 Å. A total of 54,378 unbinned sub-particles were re-extracted for CTF refinement, and finally, we achieved the 5.1 Å reconstruction on a 2-fold block. For calculation of the 3-fold block, 1,050,900 sub-particles were extracted and binned 2x. According to nonalignment 3D classification, 203,538 sub-particles were selected and locally refined to 5.1 Å. We still tried the isSPA sorting algorithms using frequencies from 1/8 $Å^{-1}$ to 1/5 $Å^{-1}$, and 97,192 sub-particles were picked according the scores, leading to a reconstruction of 4.8 Å. CTF refinement was performed using Relion, and the resolution was improved to 4.7 Å. The unbinned (1.08 Å /pixel) sub-particles were then re-extracted, and a second-round isSPA sorting (at a frequency range of 1/8 $Å^{-1}$ to 1/4.7 $Å^{-1}$) was applied. Combined with CTF refinement, 45,407 sub-particles were finally used for the 4.5 Å reconstruction.

Model building and refinement

The electron density maps of SFTSV were evaluated by FSC calculation. The overall distribution of the FSC curve is normal, indicating that there are no problems such as overfitting in the optimization of the model and the results of the electron density maps are credible. We also evaluated the local resolution of the final reconstruction. Although the resolution is still limited in some regions, the maps of most of the important regions have provided enough information on the Cα backbone and details of the side chains to allow reconstruction of the glycoprotein shell of the entire viral particle. Some crystal structures (PDB ID: 5Y10, 5G47, 4HJC and 3N43) were used as templates to fit the density map of the SFTSV glycoprotein shell with UCSF Chimera (Pettersen et al., 2004). It is well known that fitting crystal structures into the cryo-EM maps of whole virions require that domain positions are adjusted. The model building calculated with sharpening (B factors) was done manually using the program Relion. The glycoproteins model was initially fitted into the density as a rigid body dimer and then the fit was optimized by fitting individual domains separately. Further model building and structure regularization was done in COOT. The model was manually adjusted in COOT (Emsley et al., 2010) to improve the local fit and update the sequence register. The completed model was further improved through iterative positional and B-factor refinement using Phenix (Afonine et al., 2012), followed by rebuilding in COOT and evaluation using Molprobity (Afonine et al., 2018). The specific structural correction parameters were adjusted as detailed in Table S1. The structural figures were prepared using ChimeraX and PyMOL (https://pymol.org/2/).

Data availability

Density maps, structure factors and atomic models that support the findings of this study have been deposited in the Electron Microscopy Data Bank (EMDB)

and in the Protein Data Bank (PDB) with the accession codes PDB: 7X6U (Gn-Gc of SFTSV 3 fold hexamer), EMD-33027 (SFTSV 3 fold hexamer) and PDB: 7X6W (Gn-Gc of SFTSV 2 fold hexamer),EMD-33029 (SFTSV 2 fold hexamer) and PDB: 7X72 (Gn-Gc of SFTSV 5 fold pentamer), EMD-33030 (SFTSV 5 fold pentamer). The data that support this work are available from the corresponding author’s upon reasonable request.

Supplementary Figures

Supplementary Figure 1

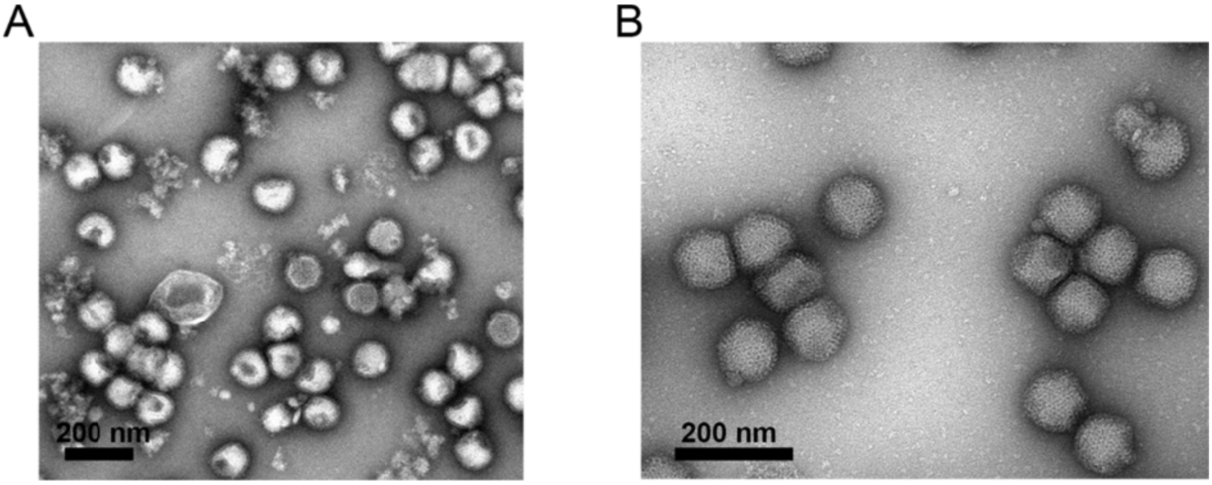

Figure. S1. Virus purification

(A) Negative-stained images of purified SFTSV particles with the traditional virus purification methods. (**B)** Negative-stained images of the good initial progress with purified SFTSV particles.

Supplementary Figure 2

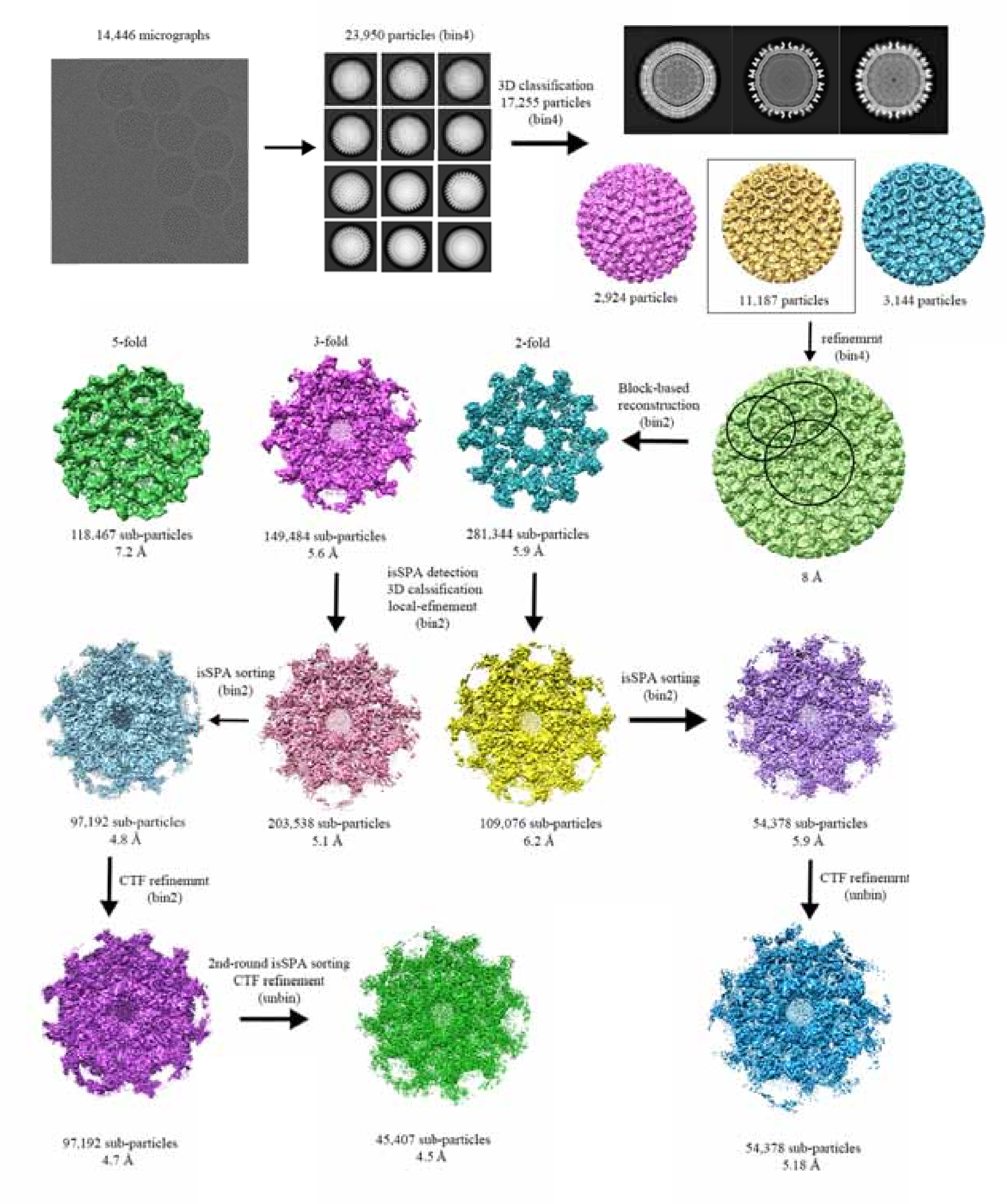

Figure. S2. Flowchart of the cryo-EM data processing

Data processing and blocked reconstructions of the SFTSV particles. Details can be found in Methods.

Supplementary Figure 3

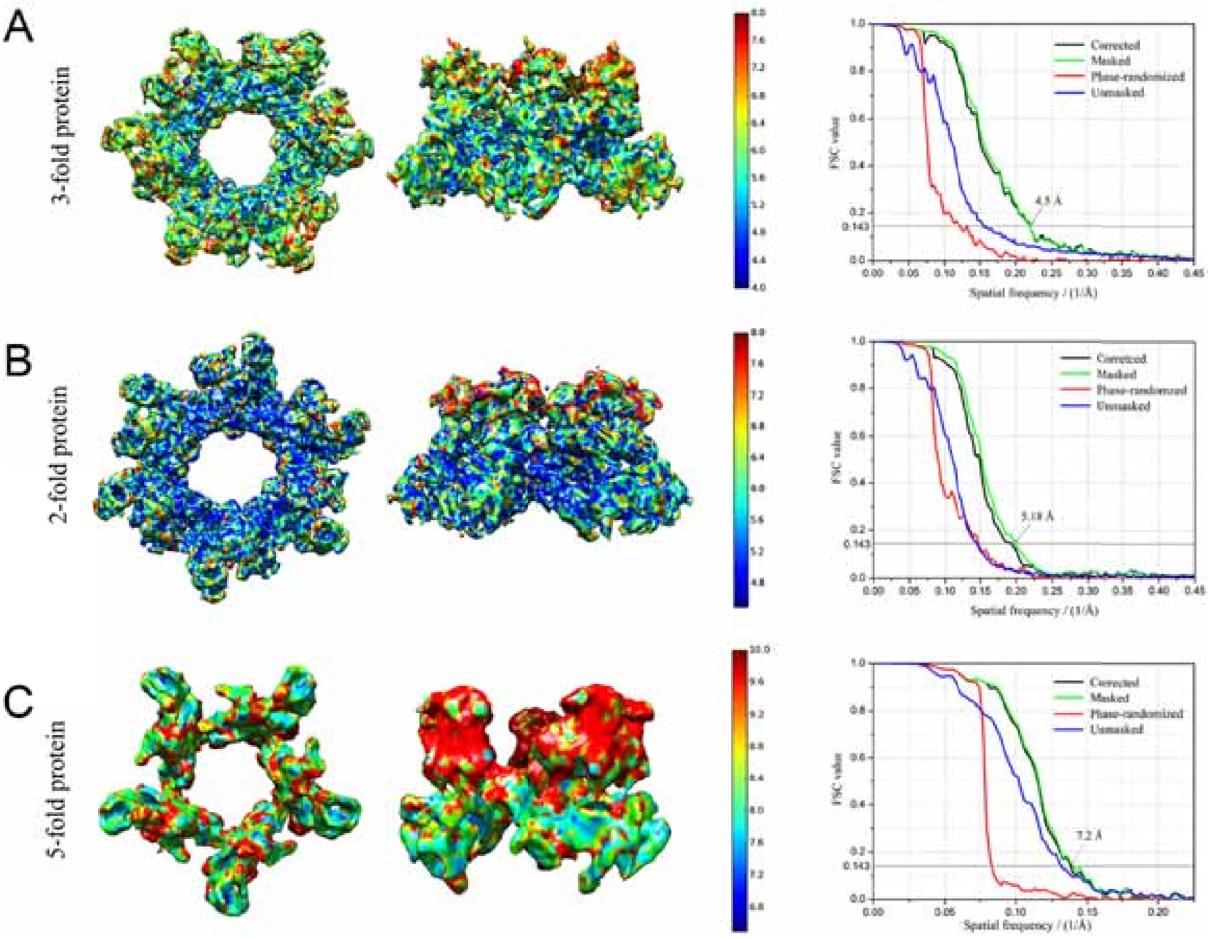

Figure. S3. Resolution assessment of the final reconstruction

(A) 3-fold protein: Local resolution distribution of the density map generated through block reconstruction and FSC curves for the final reconstruction and model-map fit evaluation. **(B)** 2-fold protein: Local resolution distribution of the density map generated through block reconstruction and FSC curves for the final reconstruction and model-map fit evaluation. **(C)** 5-fold protein: Local resolution distribution of the density map generated through block reconstruction and FSC curves for the final reconstruction and model-map fit evaluation.

Supplementary Figure 4

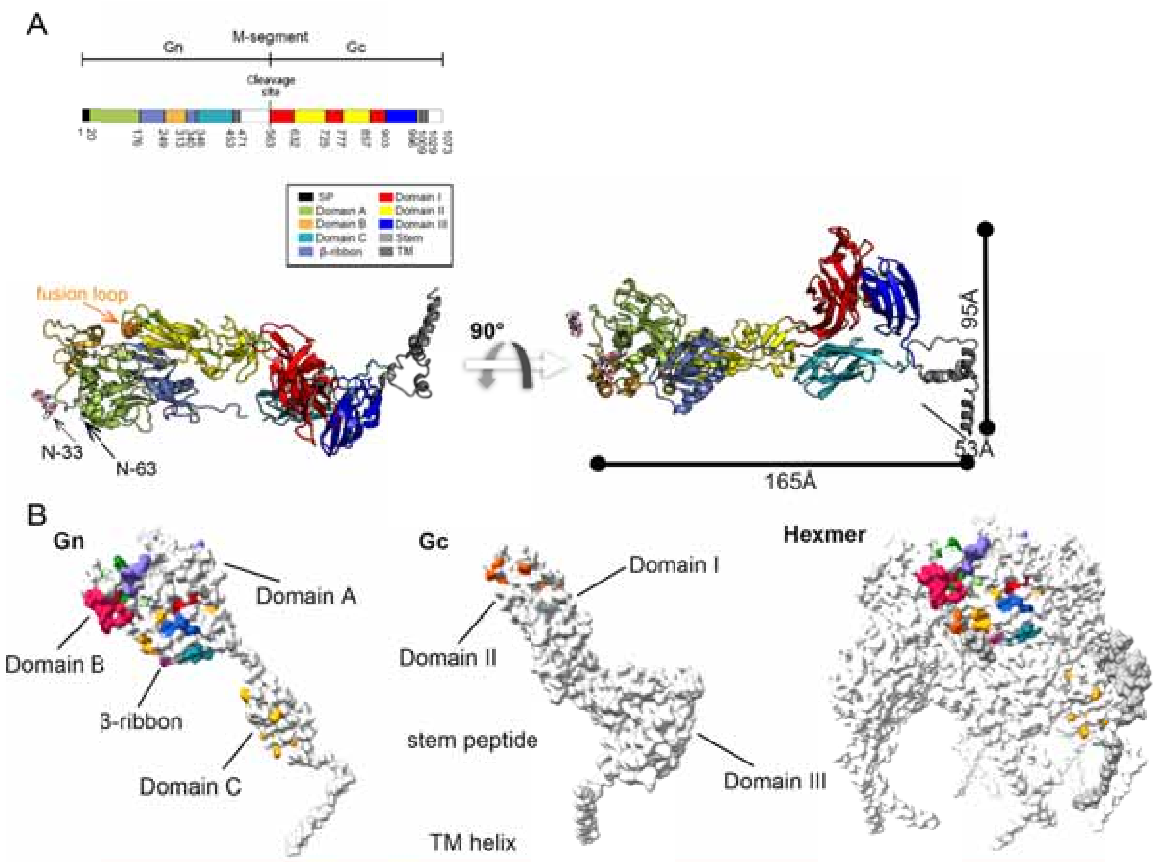

Antibody/Epitopes	Color	Amino acids
MAb4-5		^{251}SSS^{253},^{256}F,^{275}KTSG^{278},^{284}M,^{286}FCKVAGCEH^{294}
Ab10		domain B and the stem region of the Gn,main amino acids:^{315}VV^{316}, ^{318}Y, ^{322}RVRPKCYGSRMM^{334}, ^{356}C, ^{360}GVRL^{363}, ^{365}T, ^{370}L, ^{376}C, ^{387}G, ^{389}K
E1(BCEs)		^{196}FSQSEFPD^{203}
E2(BCEs)		^{232}GHSHKII^{238}
E3(BCEs)		^{256}VCYKEGTGPC^{265}
E4(BCEs)		^{316}SYGGM^{320}
RVFV antigenic patch A		^{173}TQEDATCK^{180},^{294}K
RVFV antigenic patch B		^{271}CPPK^{274}
RVFV antigenic patch C		^{223}KADPPSCD^{230}
RVFV epitopes group A		domain A and domain B of the Gn
RVFV epitopes group B		near the fusion loop of domain II of the Gc
RVFV epitopes group C		^{777}C,^{792}R,^{812}N-K^{813},^{819}G,^{821}W,^{823}C,^{825}C
RVFV epitopes group D		domain I of the Gc

Figure. S4. Position of related epitopes on the 3D structure of SFTSV

(A) Atomic models of glycoproteins from different views, colored by domains. The common feature of the class-II fusion glycoprotein architecture is the hydrophobic fusion loop located at the tip of domain II, colored orange. As determined from the crystal structure, glycoproteins contain a total of 5 N-linked glycosylations. However, in our electron density map, only two N-linked glycosylations of Gn can be seen (N33 and N63), which show atomic

structures, and three N-linked glycosylations of Gc are missing. **(B)** The currently reported phlebovirus-related antibodies or epitopes presented separately in our structures, colored by epitopes.

Supplementary Figure 5

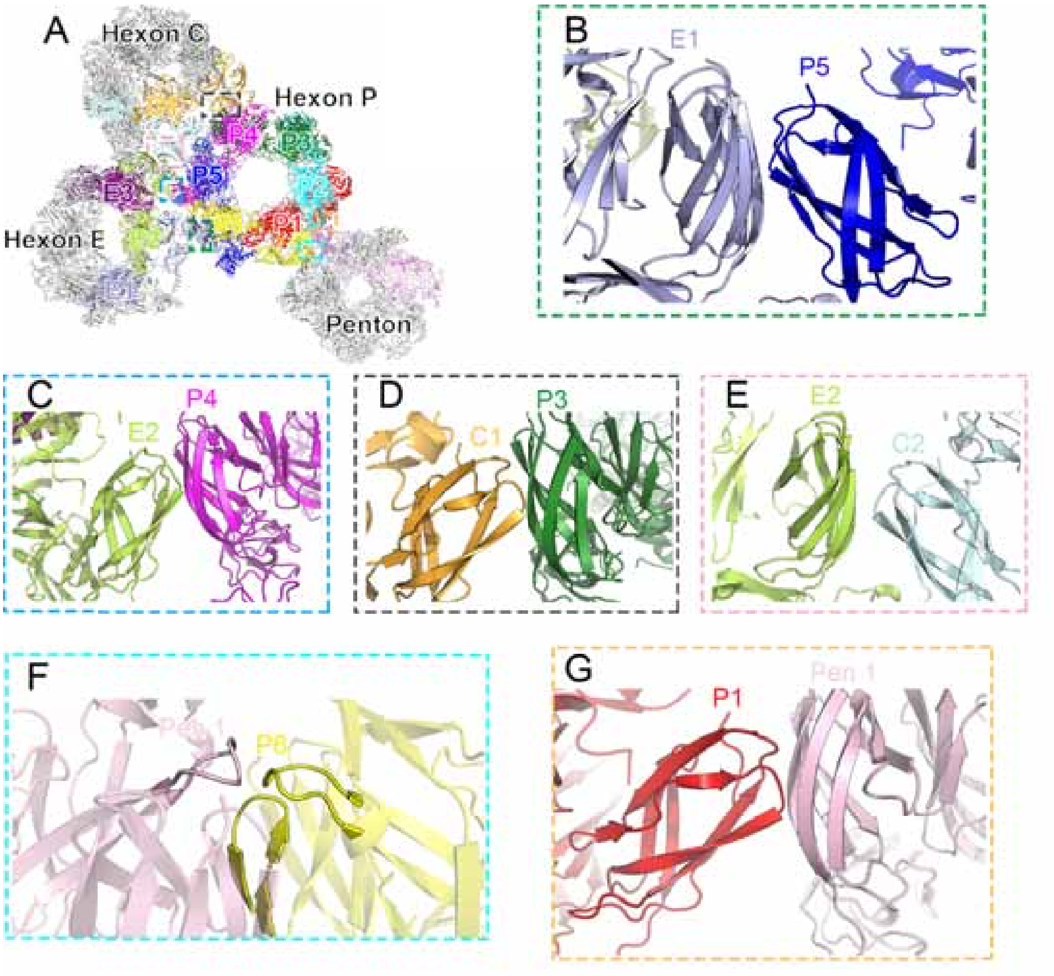

Figure. S5. Inter-capsomer contacting networks

(A) Overview of capsomer organization and interaction network within an ASU. The unique G protein conformers involved in inter-capsomer contacts are shown in different colors, and the remaining parts are shown in gray for clarity. **(B)** - **(G)** Close-up views of interactions between adjacent capsomers at different sites as indicated in **(A)**. The critical motifs or domains involved in the interactions are shown in solid colors, and the remaining parts in the background are transparent for clarity.

Supplementary Figure 6

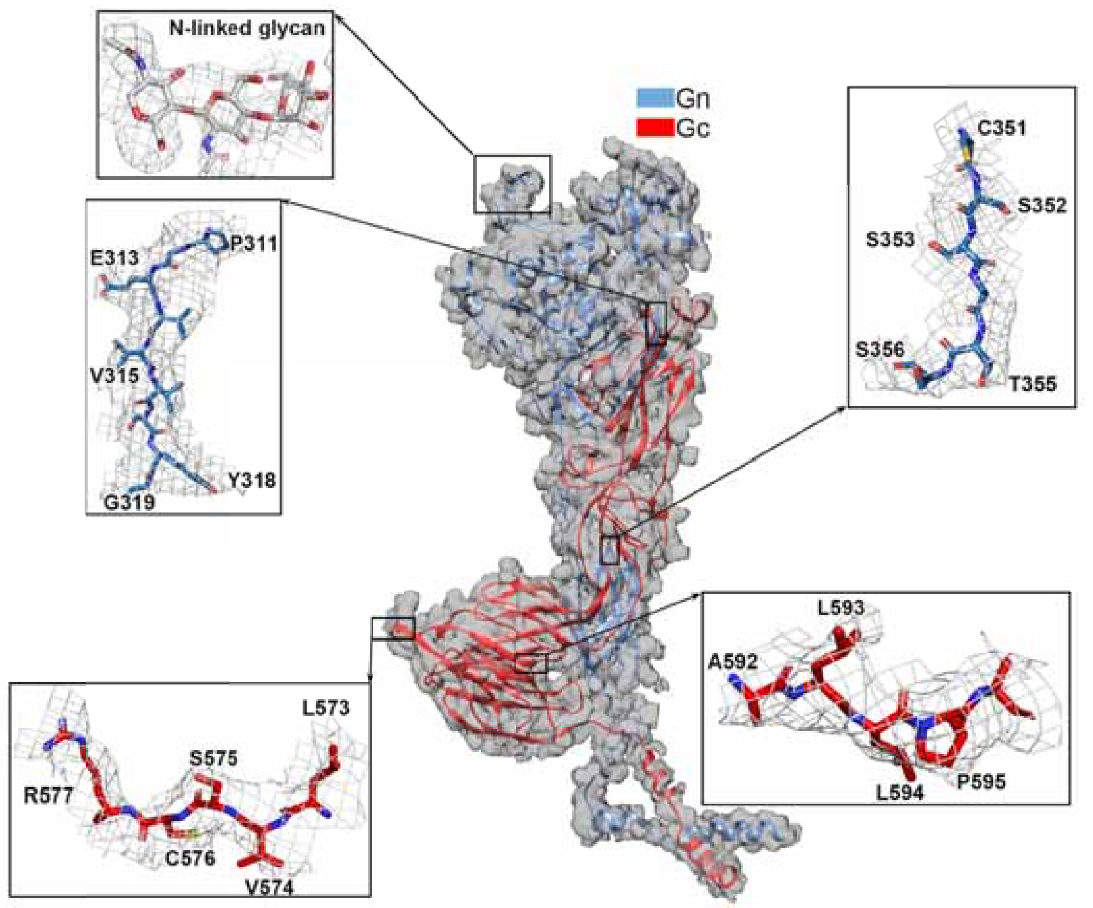

Figure. S6. Representative density maps

A shaded surface representation of the density map of the Gn-Gc prefusion heterodimer. The cartoon model of the Gn-Gc prefusion heterodimer is superimposed with a semitransparent surface. In the surrounding boxes, atomic models shown as either sticks are superimposed to indicate the representative regions in wireframes. In the stick models, amino acid residue numbers are indicated. Gn colored sky blue, Gc colored red.

Supplementary Figure 7

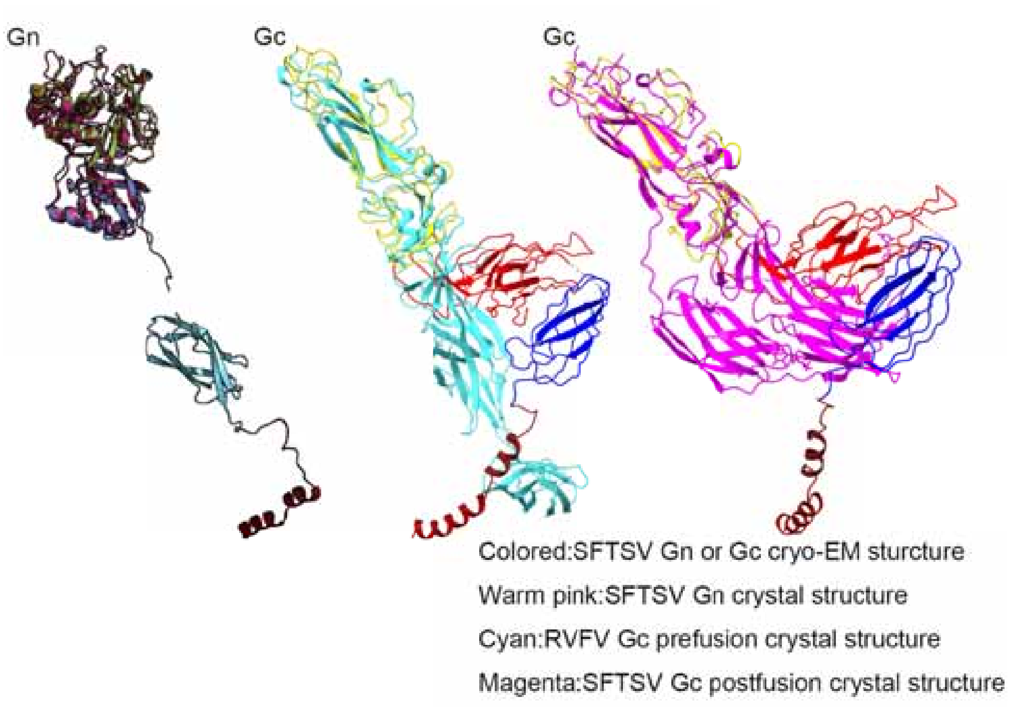

Figure. S7. Structural comparisons between the crystal structure and the cryoEM structure

Structural alignment of our cryo-EM structure to the crystal structures of SFTSV glycoproteins, and the crystal structure of RVFV Gc were done by using the Pymol program and ChimeraX.

Supplementary Tables

Supplementary Table 1. Cryo-EM data processing and refinement of models.

	SFTSV
**Data collection**	
Microscope	FEI Titan Krios
Camera	Gantan K2
Magnification	130,000
Voltage (kV)	300
Total dose ($e^{-}/Å^{2}$)	50
Defocus range (μm)	-2.5 to -2.0
Pixel size (Å/pixel)	1.08
Symmetry imposed	I3
Initial particle images (no.)	14,458
Final particle images (no.)	14,446

	3-fold Hexamer	2-fold Hexamer	Pentamer
**Data processing**			
EMDB	EMD-33027	EMD-33029	EMD-33030
Block symmetry imposed	C1	C1	C1
Map resolution (Å)	4.5	5.18	7.2
FSC threshold	0.143	0.143	0.143
Map resolution range (Å)	4.0 - 8.0	4.8 - 8.0	6.8 - 10.0
Map sharpening *B* factor (Å^2)	-150	-150	-150
Model resolution (Å)	4.5	5.18	7.2
FSC threshold			
Model resolution range (Å)	∞ to 4.5	∞ to 5.18	∞ to 7.2
Model composition			
Non-hydrogen atoms	6491	6491	6491
Protein residues	922	922	922
Ligands	0	0	0
R.m.s. deviations			
Bond lengths (Å)	0.007	0.015	0.019
Bond angles (°)	0.944	1.333	1.176
Validation			
MolProbity score	2.91	2.87	3.68
Clashscore	8.93	6.93	7.91
Poor rotamers (%)	0.06	0.06	0.81
Ramachandran plot			
Favored (%)	92.68	90.95	89.16
Allowed (%)	7.14	8.9	10.73
Disallowed (%)	0.18	0.15	0.11

Supplementary Table 2. Inter-protomer interactions in Gn-Gc prefusion heterodimer.

Molecules in Protomer A	Residue	Molecules in Protomer B	Residue
Gn	T245	Gc	S646, R648
	W247		R648, C650
	S88		S670
	Y89		D671
	Y89, H243, Y328		D672
	E242		R643
	H243		T645, D672
	G387		P599

Table. S2. The salt bridges are marked in red.

Supplementary Table 3. Inter-protomer interactions of a hexon.

Molecule/Chain	Residue	Molecule/Chain	Residue
Gn/A	S220, L221, P222	Gn/C	K288, C287
	E154, L155, C156, S157		E297, A298, S299, E300, E296, G295
	E151, S152, G153		R304, A301, K302
Gc/B	K68, S70	Gc/D	E271, L272
	K258, K257, D259		A139, P141
	E251, E250, S252, S249, S248		A132, A133, G131, G130, C129, G128
Gn/A	G319, G320	Gc/L	F195, K196, G197, V198
	S196, F197, Q199, S200		S327, S329
Gn/A	A298	Gn/K	K147
	R281, G282, D283, M284		E154, G153, L155, C156
Gc/B	Y280, S281, E271	Gc/L	S70, K68, K69
	E220		R29
	D127		S249, E250, E251, S252
	C129		E251
	H154		R325
	C125, C98		K258

Table. S3. The salt bridges are marked in red.

Supplementary Table 4. Inter-protomer interactions of a penton.

Molecule/Chain	Residue	Molecule/Chain	Residue
Gc/B	S72	Gc/D	Y280
	P191		T284

**Gc/B**	Q193, L203, T192	**Gc/J**	S285
	G28		H154
	K69		E271
	Q193		R79
	R79, S285		Q193, T192, L203
	E271		K69
	Y280		S72
	T284		P191
**Gn/A**	N214	**Gc/J**	R15

Table. S4. The salt bridges are marked in red.

Supplementary Table 5. Interactions between capsomers.

Capsomer/Molecule/Chain	Residue	Capsomer/Molecule/Chain	Residue
**C-Hex/Gn/A**	S399	**P-Hex/Gc/T**	V423
	Q348		S396
	C349		K368
**E-Hex/Gc/J**	K368	**P-Hex/Gn/O**	S372, C349
	C346		T374
	S396		Q348
**E-Hex/Gn/E**	Q422	**P-Hex/Gc/R**	L400
	C349, S1321		E349, K368
**C-Hex/Gn/C**	L400	**E-Hex/Gc/F**	V350
	S372		K368
**Penton/Gc/X**	H419	**P-Hex/Gc/N**	K10
	E420		K10
	A47, P48		S50
**Penton/Gc/X**	S396	**P-Hex/Gn/K**	Q348
	E349		V375
	K368		C349

Table. S5. The salt bridges are marked in red.

References

Chapman, N.S., Zhao, H., Kose, N., Westover, J.B., Kalveram, B., Bombardi, R., Rodriguez, J., Sutton, R., Genualdi, J., LaBeaud, A.D., *et al.* (2021). Potent neutralization of Rift Valley fever virus by human monoclonal antibodies through fusion inhibition. Proc Natl Acad Sci U S A 118.

Crowe, J.E., Jr. (2017). Principles of Broad and Potent Antiviral Human Antibodies: Insights for Vaccine Design. Cell Host Microbe 22, 193-206.

Dong, Y., Liu, Y., Jiang, W., Smith, T.J., Xu, Z., and Rossmann, M.G. (2017). Antibody-induced uncoating of human rhinovirus B14. Proc Natl Acad Sci U S A 114, 8017-8022.

Kim, K.H., Kim, J., Ko, M., Chun, J.Y., Kim, H., Kim, S., Min, J.Y., Park, W.B., Oh, M.D., and Chung, J. (2019). An anti-Gn glycoprotein antibody from a convalescent patient potently inhibits the infection of severe fever with thrombocytopenia syndrome virus. PLoS Pathog 15, e1007375.

Li, X., Mooney, P., Zheng, S., Booth, C.R., Braunfeld, M.B., Gubbens, S., Agard, D.A., and Cheng, Y. (2013). Electron counting and beam-induced motion correction enable near-atomic-resolution single-particle cryo-EM. Nat Methods 10, 584-590.

Mindell, J.A., and Grigorieff, N. (2003). Accurate determination of local defocus and specimen tilt in electron microscopy. J Struct Biol 142, 334-347.

Moming, A., Shi, S., Shen, S., Qiao, J., Yue, X., Wang, B., Ding, J., Hu, Z., Deng, F., Zhang, Y., *et al.* (2021). Fine mapping epitope on Glycoprotein-Gn from Severe Fever with Thrombocytopenia Syndrome Virus. PLoS One 16, e0248005.

Scheres, S.H. (2012). RELION: implementation of a Bayesian approach to cryo-EM structure determination. J Struct Biol 180, 519-530.

Wang, K., Zheng, B., Zhang, L., Cui, L., Su, X., Zhang, Q., Guo, Z., Guo, Y., Zhang, W., Zhu, L., *et al.* (2020). Serotype specific epitopes identified by neutralizing antibodies underpin immunogenic differences in Enterovirus B. Nat Commun 11, 4419.

Wang, Q., Ma, T., Wu, Y., Chen, Z., Zeng, H., Tong, Z., Gao, F., Qi, J., Zhao, Z., Chai, Y., *et al.* (2019). Neutralization mechanism of human monoclonal antibodies against Rift Valley fever virus. Nat Microbiol 4, 1231-1241.

Wu, Y., Zhu, Y., Gao, F., Jiao, Y., Oladejo, B.O., Chai, Y., Bi, Y., Lu, S., Dong, M., Zhang, C., *et al.* (2017). Structures of phlebovirus glycoprotein Gn and identification of a neutralizing antibody epitope. Proc Natl Acad Sci U S A 114, E7564-E7573.

Yu, X.J., Liang, M.F., Zhang, S.Y., Liu, Y., Li, J.D., Sun, Y.L., Zhang, L., Zhang, Q.F., Popov, V.L., Li, C., *et al.* (2011). Fever with thrombocytopenia associated with a novel bunyavirus in China. N Engl J Med 364, 1523-1532.

Zheng, Q., Jiang, J., He, M., Zheng, Z., Yu, H., Li, T., Xue, W., Tang, Z., Ying, D., Li, Z., *et al.* (2019). Viral neutralization by antibody-imposed physical disruption. Proc Natl Acad Sci U S A.
